# Fertility decision and its associated factors in Sub-Saharan Africa: a multilevel multinomial logistic regression analysis

**DOI:** 10.1186/s12905-022-01920-w

**Published:** 2022-08-08

**Authors:** Achamyeleh Birhanu Teshale, Misganaw Gebrie Worku, Getayeneh Antehunegn Tesema

**Affiliations:** 1 Department of Epidemiology and Biostatistics, Institute of Public Health, College of Medicine and Health Sciences, University of Gondar, Gondar, Ethiopia; 2 Department of Human Anatomy, College of Medicine and Health Science, School of Medicine, University of Gondar, Gondar, Ethiopia

**Keywords:** Fertility decisions, Fertility desire, Multinomial regression, Sub-Saharan Africa

## Abstract

**Background:**

Fertility desire is one of the predictors of contraceptive behavior and fertility-related outcomes. However, information is scarce on individual and community-level factors of women’s fertility decisions in sub-Saharan Africa.

**Objective:**

To assess fertility decisions and their associated factors in Sub-Saharan Africa.

**Methods:**

The 35 Sub-Saharan African country’s most recent demographic and health surveys (DHS) data conducted from 2008 to 2020 was used. A total of 284,744 (weighted) married women were used for analysis. The proportion of fertility decisions with their 95%CI was estimated. To assess the factors associated with fertility decisions, both random effect and fixed effect analyses were conducted. In the fixed analysis, particularly in the multivariable analysis, adjusted relative risk ratio (aRRR) with its 95% confidence interval (CI) was reported and variables with a *p*-value < 0.05 were considered significant predictors of fertility decisions.

**Results:**

In this study, 64.35% (95%CI: 64.2%, 64.5%) of the study participants had fertility desire. However, 5.4% (95%CI: 5.3, 5.5) of the study participants had undecided fertility behavior. In the multivariable analysis, desire for more children and undecided fertility desire were relatively lower among older women, women with primary, secondary, and higher education, working women, women who currently use contraceptives, women with a higher number of living children, women with higher parity, women from eastern and southern Africa, and women from wealthy households. While, the ideal number of children, women who had decision-making autonomy, and women from the rural residence were all associated with a relatively higher desire for more children and undecided fertility desire. Furthermore, respondents' education and sex of household head were associated with the desire for more children while media exposure was associated with undecided fertility desire.

**Conclusion:**

In this study, around two-thirds of women had a desire for more children and only 5.4% of women had undecided fertility desires. Both individual and community-level factors were associated with both desires for more children and undecided fertility desires. As a result, the aforementioned factors should be considered while developing reproductive health programs.

## Background

According to the United Nations report, the global human population in the year between 2020 and 2100 may increase from 7.8 billion to 10.9 billion [1]. Around 78 million people are added to the world's population each year [2] and Sub-Saharan Africa (SSA) accounts for more than half of the global fertility with an estimated fertility rate of 4.8 [3, 4]. Studies revealed that economies, food production, the general environment, and the global climate will be greatly affected if the current population is increased by 40% [5–7]. Rapid population growth puts more pressure on already strained resources and poses a great challenge for sustainable development. Africa is responsible for greater than 50% of the projected increase in the global population by 2050. Countries of SSA, with a projected addition of greater than one billion people, account for more than 50% of the growth of the world's population in the year between 2019 and 2050 [8].

Contraceptive use is essential to achieving fertility desire and pregnancy spacing [9]. However, it is considered to be low in SSA [10]. Besides, in many SSA countries, more than half of women with a higher number of children still desire to have more children [11].

According to different studies, both individual and community levels factors such as age, marital status, income, educational level, parity, and residence are associated with fertility decisions. Besides, vast empirical evidence has confirmed that fertility decisions have a great variation across countries [12–16].

One of the predictors of contraceptive behavior and reproductive-related outcomes is fertility decision and understanding its magnitude has practical implications for developing family planning programs and, more broadly, for achieving the sustainable development goal [17–19]. Therefore, this study aimed to assess fertility decisions and associated factors in sub-Saharan Africa. Assessment of fertility desire is a very important issue to be considered and understanding the factors for the extraordinary population growth in SSA is critical for many aspects of international and national planning [20]. Besides, findings from such a multicountry study will be vital to strengthening existing measures to tackle high fertility and improvement in maternal and child health.

## Methods

### Study design and population

**Fig. 1:**
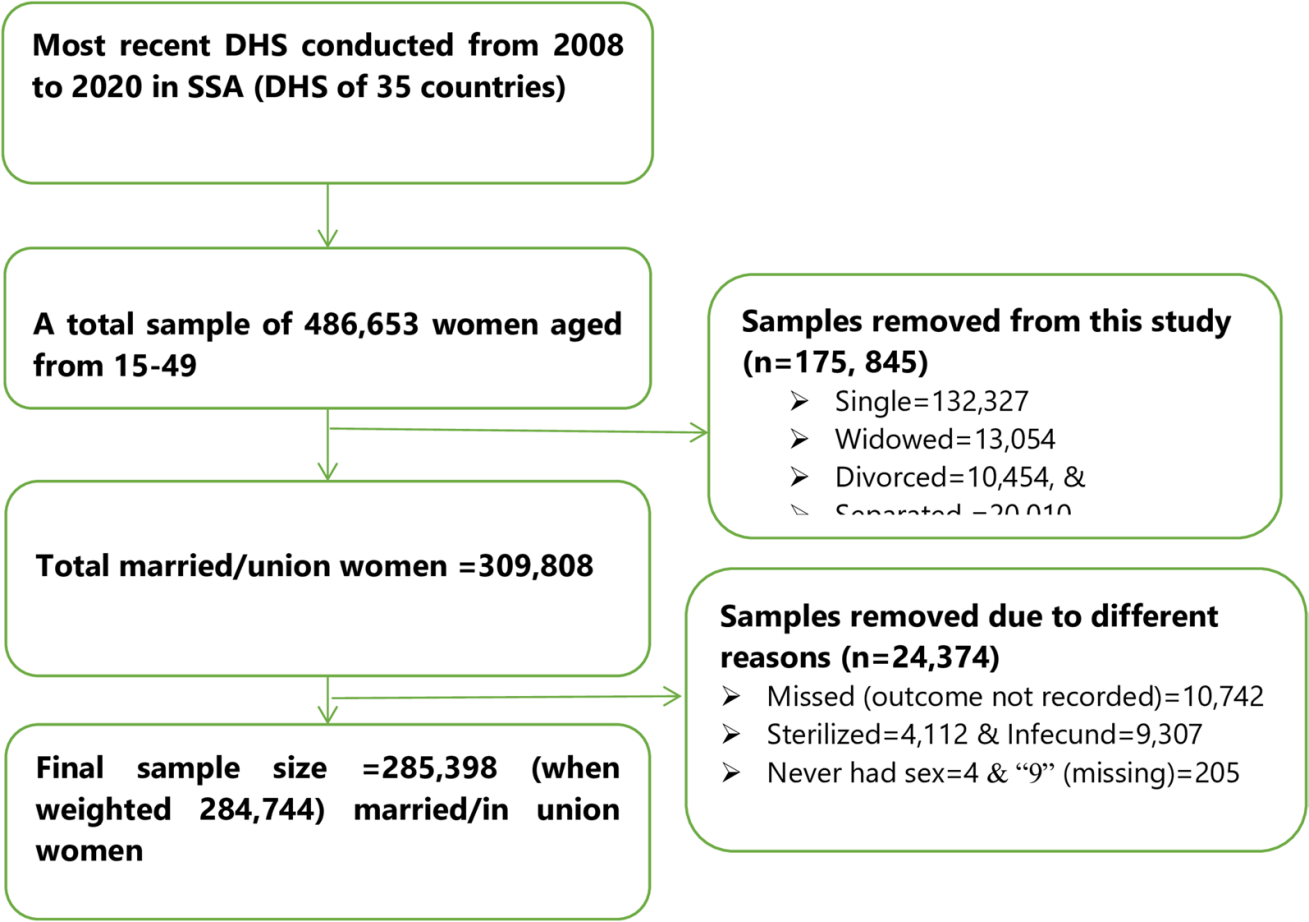
Schematic presentation of sample extraction

The most recent demographic and health surveys (DHS) data from 35 SSA countries, conducted between 2008 and 2020, were used. These DHS used a two-stage stratified sampling technique. A total of 284,744 (weighted) married women who had complete information on fertility decisions were used for this study (Fig. 1, Table 1). Details of the DHS methodology are reported elsewhere [21].

**Table 1:** A detailed description of the study sample used for the study

Country	Unweighted frequency (N = 285,398)	Percentage (%)	Weighted frequency (N = 284,744)	Percentage (%)
Western Africa	121,769	42.67	121,052	42.51
Burkina Faso	12,953	4.54	13,127	4.61
Benin	10,789	3.78	10,794	3.79
Cote d’Ivoire	6191	2.17	6048	2.12
Ghana	5177	1.81	5035	1.77
Gambia	7908	2.77	7370	2.59
Guinea	7325	2.57	7266	2.55
Liberia	4502	1.58	4077	1.43
Mali	7860	2.75	8143	2.86
Nigeria	28,035	9.82	28,341	9.95
Niger	9210	3.23	9591	3.37
Sierra Leone	9437	3.31	9339	3.28
Senegal	6247	2.19	5877	2.06
Togo	6135	2.15	6046	2.12
Eastern Africa	103,256	36.18	104,218	36.60
Burundi	9344	3.27	9578	3.36
Ethiopia	9558	3.35	10,017	3.52
Kenya	8623	3.02	8325	2.92
Comoros	3040	1.07	2988	1.05
Madagascar	11,559	4.05	11,697	4.11
Malawi	14,063	4.93	14,104	4.95
Mozambique	8551	3.00	8845	3.11
Rwanda	6725	2.36	6820	2.4
Tanzania	7748	2.71	7741	2.72
Uganda	10,815	3.79	10,654	3.74
Zambia	7328	2.57	7399	2.6
Zimbabwe	5902	2.07	6050	2.12
Central Africa	51,296	17.97	50,431	17.71
Angola	7594	2.66	7519	2.64
DR Congo	11,847	4.15	11,534	4.05
Congo	6282	2.20	5898	2.07
Cameroon	6997	2.45	7298	2.56
Gabon	4429	1.55	4228	1.49
Sao Tome & Principe	1705	0.60	1674	0.59
Chad	12,442	4.36	12,280	4.31
Southern Africa	9,077	3.18	9,043	3.18
Lesotho	3505	1.23	3505	1.23
Namibia	3022	1.06	2816	0.99
South Africa	2550	0.89	2722	0.96

### Study variables

#### Outcome variable

The outcome variable was fertility decisions. Women were asked whether they want to have a child in the future with the following options: have a desire for more children, have no desire for more children, undecided fertility desire, declared sterilized, and declared infecund. Women who declared infecund, sterilized, and with missing information were excluded because their responses were unclear about their fertility decisions. Finally, fertility decision was computed on the three responses (those who have a desire for more children, have no desire for more children, and undecided fertility desire). Then, no desire was coded as 0, desire for more children was coded as 1, and undecided fertility desire was coded as 2.

#### Independent variables

Independent variables were grouped into the individual-level and community-level factors. The individual-level factors included respondent age, respondent level of education, husband level of education, parity, exposure to media, current use of contraceptives, the ideal number of children, decision-making autonomy, number of living children, women employment, sex of household head, and wealth status. The community-level factors were place of residence and region of Africa (particularly SSA).

#### Operational definition

##### Media exposure

Constructed from three variables (frequency of listening to the radio, watching television, and reading newspaper/magazine). For this study, it was recoded into yes (if women were exposed to at least one media) and no (otherwise).

##### Decision-making autonomy

It was constructed from three variables (decision on respondent's healthcare, decision on large household purchase, and decision on visits to family or relatives) and recoded into "respondents alone" if a woman is the decision maker and "otherwise" if another person (husband, relatives, or friends) is the decision-maker [16].

#### Data management and statistical analyses

STATA version 16 was used for data management (extraction, recoding, and cleaning) and statistical analyses (descriptive and analytical analysis). All analyses were weighted to make the data representative, to account for the non-response rate, and to get a better statistical estimate [22]. The proportion of fertility decisions with their 95%CI was estimated and to assess the factors associated with fertility decisions, both random effect and fixed effect analyses were employed.

##### Fixed effect analysis

We employed a multilevel multinomial logistic regression analysis. While doing the analysis, we have fitted four models; null model, model 1, model 2, and model 3. The null model was fitted with only the outcome variable. Model 1, model 2, and model 3 were fitted using individual-level variables, community-level variables, and both individual and community-level variables respectively. To select eligible variables for the multilevel analysis, a bivariable multilevel multinomial regression was fitted first and those variables with a p-value less than 0.20 in the bivariable analysis were considered eligible. Then, in the multivariable analysis, the adjusted relative risk ratio (aRRR) with its 95% confidence interval (CI) was reported. Finally, variables with a *p*-value < 0.05 were considered significant predictors of fertility decisions.

##### Random-effect analysis

It was conducted to assess the cluster level variability of fertility decisions. Intraclass correlation coefficient (ICC) and proportional change in variance (PCV) were calculated. Log-likelihood and deviance were used to verify model fitness, and a model with the highest log-likelihood and lowest deviance has been deemed as a best-fit model.

## Results

### Background characteristics of respondents

A total weighted sample of 284,744 married women was used for the final analysis. The mean age of the study participants was 30.82 (SD ± 8.29) years. Around two-thirds (65.74%) of the study participants were employed (workers). Most (84.99%) of women were from male-headed households and only 5.21% of women had decision-making autonomy. Around one-half, (51.73%) of women had 1–3 living children and about two-thirds of women had exposure to at least one media (Table 2).

**Table 2:** Sociodemographic characteristics of respondents

Variables	Frequency (N = 284,744)	Percentage (%)
*Age (years)*		
15–19	20,858	7.33
20–24	50,688	17.80
25–29	63,321	22.24
30–34	54,459	19.13
35–39	44,900	15.77
40–44	30,223	10.61
45–49	20,295	7.13
*Respondent educational status*		
No formal education	112,304	39.44
Primary education	92,193	32.38
Secondary education	68,483	24.05
Higher	11,764	4.13
*Husband educational status*		
No formal education	101,529	35.66
Primary education	79,684	27.98
Secondary education	81,114	28.49
Higher	22,417	7.87
*Women employment*		
Not Working	97,563	34.26
Working	187,181	65.74
*Sex of household head*		
Male	242,015	84.99
Female	42,729	15.01
Decision-making autonomy		
Respondent alone	14,842	5.21
Otherwise	269,902	94.79
*Number of living children*		
0	22,865	8.03
1–3	147,306	51.73
4 & above	114,573	40.24
Parity		
Null	20,164	7.08
One	42,923	15.07
Two	48,212	16.93
Three	43,478	15.27
4 & above	129,967	45.64
*Ideal number of children*		
0–3	51,919	18.23
4–5	106,923	37.55
6 & above	109,023	38.29
Non-numeric response	16,879	5.93
*Media exposure*		
No	94,186.27	33.08
Yes	190,558.1	66.92
*Wealth status*		
Poorest	55,765	19.58
Poorer	57,711	20.27
Middle	56,565	19.87
Richer	57,388	20.15
Richest	57,315	20.13
*Current contraceptive use*		
No	204,379	71.78
Yes	80,365	28.22
Residence		
Urban	97,098	34.10
Rural	187,646	65.90
*Region of SSA*		
Western	121,052	42.51
Eastern	104,218	36.60
Central	50,431	17.71
Southern	9043	3.18

### Proportion of fertility decisions in SAA

**Fig. 2:**
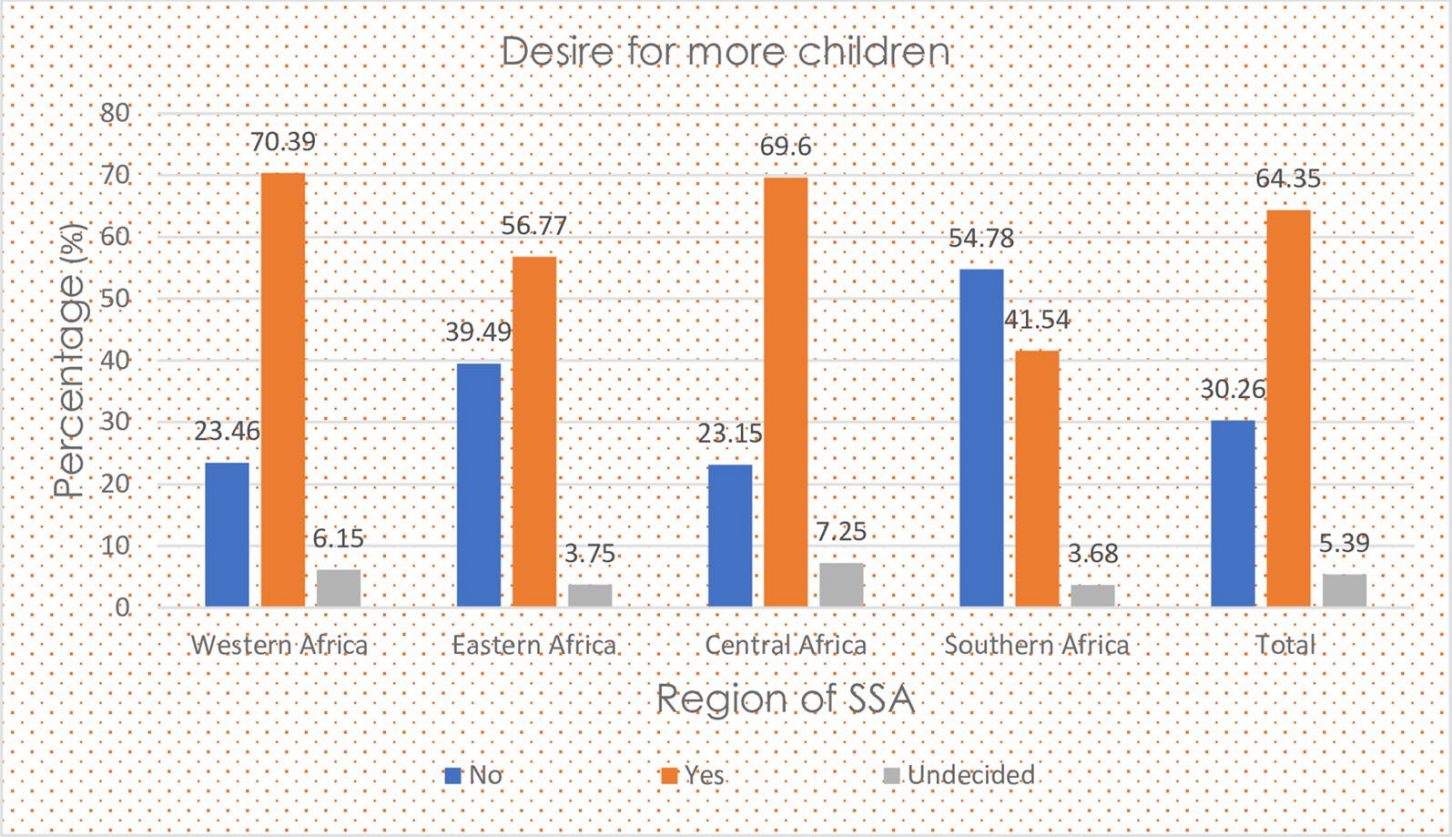
Proportion of fertility decisions by SSA region

In this study, 64.35% (95%CI: 64.2, 64.5) and 5.4% (95%CI: 5.3, 5.5) of the study participants desire more children and had undecided/undetermined fertility desires, respectively. Besides, fertility decisions had significant variation between countries and African regions (Table 3, Fig. 2).

**Table 3:** Fertility preference by country of SSA

Country	Fertility preference/desire for more children		
	No	Yes	Undecided
Angola	2475	3965	1079
	32.91	52.74	14.35
Burkina Faso	3196	9677	254
	24.35	73.71	1.94
Benin	2718	7153	923
	25.18	66.27	8.55
Burundi	4394	5087	97
	45.88	53.11	1.01
DR Congo	2730	8281	523
	23.67	71.80	4.53
Congo	1113	4374	411
	18.86	74.17	6.97
Cote d’Ivoire	1291	4500	257
	21.35	74.40	4.24
Cameroon	1872	5084	342
	25.65	69.67	4.69
Ethiopia	3713	5775	529
	37.07	57.65	5.28
Gabon	1011	2865	352
	23.93	67.75	8.33
Ghana	1873	2737	425
	37.21	54.36	8.44
Gambia	1273	5833	264
	17.28	79.14	3.58
Guinea	1351	5267	648
	18.60	72.49	8.92
Kenya	4090	3966	269
	49.13	47.65	3.23
Comoros	617	2247	124
	20.65	75.21	4.14
Liberia	1431	2269	377
	35.09	55.65	9.25
Lesotho	2024	1439	42
	57.75	41.05	1.21
Madagascar	4989	6399	309
	42.65	54.71	2.64
Mali	1724	6069	350
	21.17	74.53	4.30
Malawi	6160	7190	754
	43.68	50.98	5.34
Mozambique	2627	5710	508
	29.70	64.56	5.74
Nigeria	7051	19,539	1751
	24.88	68.94	6.18
Niger	828	8525	238
	8.64	88.88	2.48
Namibia	1415	1261	140
	50.25	44.78	4.97
Rwanda	3295	3432	93
	48.32	50.32	1.36
Sierra Leone	2498	5418	1423
	26.74	58.02	15.24
Senegal	1150	4638	89
	19.57	78.93	1.50
Sao Tome and Principe	876	740	58
	52.35	44.21	3.44
Chad	1596	9791	893
	13.00	79.73	7.27
Togo	2012	3588	446
	33.28	59.34	7.38
Tanzania	2113	5359	269
	27.29	69.23	3.48
Uganda	3940	6383	331
	36.98	59.91	3.11
South Africa	1515	1057	150
	55.65	38.82	5.53
Zambia	2749	4251	399
	37.15	57.46	5.40
Zimbabwe	2463	3364	223
	40.71	55.61	3.68

The first row has *frequencies* and the second row has *row percentages*

### Factors associated with fertility decisions in SSA

All variables in the bivariable analysis had *p* < 0.20 and were eligible for the multivariable analysis. In the multivariable multilevel multinomial analysis, age of the respondent, husband's education, employment, sex of household head, decision-making autonomy, number of living children, parity, the ideal number of children, wealth status, residence, and region of Africa were associated with both desires for more children and undecided fertility desire. Respondent’s education and sex of household head were associated with the desire for more children while media exposure was associated with undecided fertility desire only (Table 4).

**Table 4:** Multivariable multilevel multinomial regression for assessing factors associated with fertility desire in SSA

Variables	Null model	Model 1		Model 2		Model 3	
		Individual-level characteristics		Community-level characteristics		Both individual and community-level characteristics	
		Desire more children aRRR (95%CI)	Undecided aRRR (95%CI)	Desire more children aRRR (95%CI)	Undecided aRRR (95%CI)	Desire more children aRRR (95%CI)	Undecided aRRR (95%CI)
*Age (years)*							
15–19		1.00	1.00			1.00	1.00
20–24		1.41 (1.26, 1.58)	0.96 (0.82, 1.12)			1.52 (1.36, 1.71)***	1.05 (0.90, 1.23)
25–29		1.37 (1.22, 1.54)	0.91 (0.78, 1.07)			1.56 (1.39, 1.76)***	1.05 (0.90, 1.24)
30–34		0.82 (0.73, 0.92)	0.69 (0.59, 0.81)			0.97 (0.86, 1.09)	0.82 (0.70, 0.97)*
35–39		0.38 (0.34, 0.43)	0.52 (0.44, 0.61)			0.45 (0.40, 0.51)***	0.61 (0.52, 0.724)***
40–44		0.14 (0.12, 0.16)	0.28 (0.24, 0.34)			0.17 (0.15, 0.19)***	0.33 (0.28, 0.40)***
45–49		0.05 (0.04, 0.06)	0.16 (0.13, 0.19)			0.06 (0.05, 0.06)***	0.19 (0.16, 0.23)***
*Respondent educational status*							
No formal education		1.00	1.00			1.00	1.00
Primary education		0.70 (0.67, 0.73)	0.76 (0.71, 0.81)			0.83 (0.80, 0.87)***	0.93 (0.87, 1.01)
Secondary education		0.77 (0.73, 0.81)	0.87 (0.80, 0.95)			0.89 (0.85, 0.94)***	0.95 (0.87, 1.04)
Higher		0.85 (0.77, 0.94)	0.86 (0.73, 1.01)			1.04 (0.94, 1.14)	1.01 (0.86, 1.19)
*Husband educational status*							
No formal education		1.00	1.00			1.00	1.00
Primary education		0.67 (0.64, 0.70)	0.58 (0.54, 0.62)			0.81 (0.78, 0.84)***	0.75 (0.69, 0.80)***
Secondary education		0.76 (0.73, 0.80)	0.85 (0.79, 0.92)			0.78 (0.74, 0.82)***	0.83 (0.77, 0.89)***
Higher		0.93 (0.86, 0.99)	0.98 (0.87, 1.10)			0.89 (0.83, 0.95)**	0.89 (0.79, 1.00)
Women employment							
Working		1.03 (0.99, 1.06)	0.88 (0.83, 0.93)			0.91 (0.88, 0.94)*	0.78 (0.73, 0.82)***
Not working		1.00	1.00			1.00	1.00
*Sex of household head*							
Male		1.00	1.00			1.00	1.00
Female		0.92 (0.88, 0.96)	1.00 (0.94, 1.07)			0.95 (0.92, 0.99)***	1.04 (0.98, 1.11)
*Decision-making autonomy*							
Respondent alone		1.00	1.00			1.00	1.00
Otherwise		1.67 (1.57, 1.78)	1.30 (1.17, 1.44)			1.59 (1.49, 1.69)***	1.25 (1.12, 1.38)***
*Number of living children*							
0		1.00	1.00			1.00	1.00
1–3		0.23 (0.17, 0.32)	0.50 (0.31, 0.79)			0.23 (0.16, 0.32)***	0.49 (0.31, 0.79)**
4 & above		0.06 (0.04, 0.09)	0.28 (0.17, 0.45)			0.06 (0.04, 0.08)***	0.28 (0.17, 0.45)***
*Parity*							
Null		1.00	1.00			1.00	1.00
One		1.58 (1.12, 2.24)	1.11 (0.69, 1.80)			1.54 (1.08, 2.19)*	1.06 (0.65, 1.73)
Two		0.41 (0.29, 0.58)	0.66 (0.41, 1.07)			0.37 (0.26, 0.53)***	0.59 (0.36, 0.95)*
Three		0.17 (0.12, 0.24)	0.48 (0.29, 0.78)			0.14 (0.10, 0.20)***	0.39 (0.24, 0.64)***
4 & above		0.14 (0.10, 0.20)	0.40 (0.24, 0.66)			0.11 (0.08, 0.16)***	0.31 (0.19, 0.52)***
*Ideal number of children*							
0–3		1.00	1.00			1.00	1.00
4–5		4.48 (4.26, 4.71)	1.63 (1.49, 1.79)			3.88 (3.68, 4.08)***	1.39 (1.27, 1.53)***
6 & above		17.93 (16.85, 19.09)	2.74 (2.46, 3.06)			14.18 (13.31, 15.11)***	2.09 (1.87, 2.34)***
Non-numeric response		13.13 (12.02, 14.35)	6.25 (5.46, 7.17)			10.40 (9.49, 11.38)***	4.71 (4.10, 5.41)***
*Media exposure*							
Yes		1.04 (1.01, 1.07)	0.75 (0.70, 0.79)			1.05 (1.00, 1.08)	0.73 (0.68, 0.78)***
No		1.00	1.00			1.00	1.00
*Wealth status*							
Poorest		1.00	1.00			1.00	1.00
Poorer		0.94 (0.90, 0.98)	0.97 (0.90, 1.04)			0.92 (0.88, 0.96)***	0.92 (0.86, 0.99)*
Middle		0.93 (0.89, 0.98)	0.96 (0.89, 1.04)			0.91 (0.86, 0.95)***	0.88 (0.82, 0.96)***
Rich		0.94 (0.89, 0.99)	1.00 (0.91, 1.09)			0.93 (0.88, 0.98)**	0.86 (0.79, 0.94)***
Richer		0.91 (0.86, 0.96)	0.99 (0.89, 1.10)			0.92 (0.86, 0.98)*	0.81 (0.73, 0.91)***
*Current contraceptive use*							
Yes		0.70 (0.68, 0.72)	0.53 (0.50, 0.56)			0.81 (0.79, 0.84)***	0.65 (0.61, 0.69)***
No		1.00	1.00			1.00	1.00
*Residence*							
Urban				1.00	1.00	1.00	1.00
Rural				1.14 (1.11, 1.18)	0.97 (0.90, 1.04)	1.22 (1.16, 1.28)***	0.86 (0.79, 0.93)***
*Region of SSA*							
Western				1.00	1.00	1.00	1.00
Eastern				0.47 (0.45, 0.48)	0.36 (0.33, 0.39)	0.47 (0.45, 0.49)***	0.37 (0.34, 0.41)***
Central				1.00 (0.95, 1.05)	1.17 (1.08, 1.28)	0.99 (0.93, 1.05)	1.06 (0.97, 1.16)
Southern				0.25 (0.24, 0.26)	0.25 (0.21, 0.29)	0.21 (0.19, 0.23)***	0.20 (0.17, 0.24)***

Women in the age group 20–24 and 25–29 had 1.52 (aRRR = 1.52; 95%CI; 1.36, 1.71) and 1.56 (aRRR = 1.56; 95%CI; 1.39, 1.76) times higher likelihood for the desire for more children respectively as compared to women in the age group 15–19 years. However, desire for more children among women in the age group 35–39, 40–44, and 45–49 was relatively 55% (aRRR = 0.45; 95%CI; 0.40, 0.51), 83% (aRRR = 0.17; 95%CI; 0.15, 0.19), and 94% (aRRR = 0.06; 95%CI; 0.05, 0.06) lower as compared to women in the age group 15–19. The desire for more children was relatively reduced by 17% (aRRR = 0.83; 95%CI; 0.80, 0.87) and 11% (aRRR = 0.89; 95%CI; 0.85, 0.94) if a woman had primary and secondary education respectively as compared to women with no formal education. Women whom their husband have primary (aRRR = 0.81; 95%CI; 0.78, 0.84), secondary (aRRR = 0.78; 95%CI; 0.74, 0.82), and higher education (aRRR = 0.89; 95%CI; 0.83, 0.95) had relatively less desire for more children. Besides, working women (aRRR = 0.91; 95%CI; 0.88, 0.94), women from male-headed households (aRRR = 0.95; 95%CI; 0.92, 0.99), those who use contraceptives (aRRR = 0.81; 95%CI; 0.79, 0.84), women who have a higher number of living children, parous women, women from eastern and southern Africa, and women from wealthy households had relatively less desire for more children. While, having a higher number of ideal children, having decision-making autonomy, and being from a rural residence were associated with a relatively higher desire for more children. Regarding factors associated with undecided fertility desire, older women, higher husband educational status, working women, a higher number of living children, higher parity, having exposure to media, women from wealthy households, women from rural residence, women from eastern and southern Africa had relatively less likelihood of undecided fertility desire. While women who had decision-making autonomy and a higher number of ideal children had relatively higher undecided future fertility desires (Table 4).

Regarding the random effect results, in the null model, there were substantial variations in fertility decisions across clusters (variance = 0.06, 95% CI; 0.04, 0.08). The null model also showed that around 2% of the total variance in fertility decisions was attributed to between-cluster variation (ICC = 0.018). Besides, the highest PCV in model 3 revealed that about 43% of the variability in fertility decisions was explained by both individual and community-level characteristics. Looking at model fitness, model 3 was the best-fitted model since it had the lowest deviance (Table 5).

**Table 5:** Random effect parameters in assessing fertility decisions in SSA

Parameter	Null model	Model 1	Model 2	Model 3
Variance (95%CI)	0.06 (0.047, 0.077)	0.037 (0.030, 0.047)	0.055 (0.041, 0.075)	0.034 (0.027, 0.044)
ICC	0.018	0.011	0.016	0.010
PCV	Reference	0.383	0.083	0.433
LL	− 228,134.84	− 159,310.85	− 222,695.65	− 156,631.29
Deviance	456,269.68	318,621.7	445,391.3	313,262.58

## Discussion

This study aimed to assess fertility decisions and their associated factors in Sub-Saharan Africa. The study at hand revealed that 64.35% and 5.4% of the study participants had a desire for more children and did not decide about their fertility, respectively. This desire for more children is comparable with studies conducted in Sub-Saharan Africa and Uganda [16, 23]. However, it was higher than study findings from elsewhere [7, 24, 25]. Discrepancies in research scope and setting, the sample population, and the time these studies were conducted are all plausible explanations for the differences in study findings. The higher proportion of desire for more children found in this study could be explained by the priority placed on having more children in most regions of SSA.

In the multilevel multinomial regression, both individual and community-level factors were associated with both desires for more children and undecided fertility desires.

Women in the young age group had a relatively higher desire for more children, however, as the women get older the relative desire for more children and undecided fertility desire were lower as compared to women in the age group 15–19. This finding is comparable with different studies conducted elsewhere [16, 26–30]. This could be because younger women did not achieve their reproductive goals and are more inclined to want more children later in life.

In this study, women with primary, secondary, and higher education had less desire to have more children as compared to those women with no formal education. This finding is in line with many previous works of literature in different settings [16, 31–33]. The possible explanation is that for highly educated women, it is sometimes problematic to combine many children and life goals such as occupying a certain managerial position. Furthermore, less educated women are usually unemployed and spend the majority of their time as housewives caring for their children, which allows them to continue to have additional children. It's also possible that the majority of women in this study live in rural areas where children are valued as assets.

We observed from this study that women from wealthy households and working women were less likely to desire more children. This corroborates with studies conducted in SSA [16] and Iran [34]. This may be due to the perception of women from higher socioeconomic levels that having more children is a burden that strains resources such as time. Women with poor socioeconomic status, on the other hand, may prefer to have more children and they perceive it as a logical economic decision because each child is seen as an additional asset for stability when they become old.

Consistent with a study finding from SSA [16], women who could not make decisions on their own were more likely to desire additional children. This indicated that making life choices without interference gives people the opportunity to practice or apply their choices. The study also revealed that women with a higher number of living children were less likely to desire more children. This is in line with a study finding from SSA [16, 30]. The possible explanation is that women with a larger number of living children may be satisfied with their current family size or have met their reproductive goals. We also noted that a higher ideal number of children was associated with a higher likelihood of desiring more children. The study conducted in sub-Saharan Africa also reported that having at least six ideal numbers of children is linked to a higher likelihood to desire more children [16].

Consistent with other study findings [16, 27, 35], contraceptive use was associated with the desire for more children. Contraception users were found to have a decreased desire to have more children than their counterparts. The study also showed that women who were from rural areas were more likely to desire more children, compared to women in urban areas. This is consistent with what is reported in Nigeria and Iran [30, 32]. This could be because women in rural areas are engaged in farming and see children as a source of labor for their agricultural activities. Moreover, consistent with different study findings [16], this study revealed that there is a regional variation in the desire for more children. This may be due to the cultural and socioeconomic differences between regions of SSA.

This study has both strengths and limitations. A major strength of this study is the use of nationally representative datasets from SSA nations and the application of relevant statistical techniques (multilevel multinomial regression analysis). Despite these advantages, there may be a possibility of social desirability bias. In addition, important confounding factors such as HIV status and other sociocultural factors that may be associated with reproductive desire are not considered. Furthermore, because no previous research on undecided fertility desire has been conducted, we are unable to discuss factors associated with undecided fertility desire in detail.

## Conclusion

Around two-thirds of women desire more children and a small proportion of women had undecided fertility desires. Both individual and community-level factors were associated with desires for more children and undecided fertility desires. Among individual-level factors, older age, having primary, secondary, and higher education, working, having decision-making autonomy, a higher number of living children, higher parity, being using contraceptives, a lower ideal number of children, and being from wealthy households were associated with a lower relative risk for the desire for more children and undecided fertility desire, respectively. Besides, being from female-headed households was associated with a relatively higher likelihood of desire for more children while women whose husbands had primary and above education and had been exposed to media had a lower likelihood of undecided fertility desire. Among community-level variables, rural residence was positively associated with a desire for more children and negatively associated with undecided fertility desire. In addition, being from the eastern and southern regions of Africa was associated with a lower risk for desiring more children and undecided fertility desire, respectively. As a result, organizations such as the United Nations Population Fund and Population Media Center should emphasize the discovered determinants while intervening in rapid population growth due to unlimited fertility desire. Besides, those aiming at reducing the fertility rate of women such as the sustainable development goal should focus on drivers of desire for more children, that is both individual and community-level factors.

## Data Availability

All result-based data are within the manuscript and anyone can access the data set from the measure DHS program using https://dhsprogram.com.

## Abbreviations

aRRR: Adjusted relative risk
CI: Confidence interval
DHS: Demographic and health surveys,
ICC: Intraclass correlation coefficient
PCV: Proportional change in variance
SDG: Sustainable development goal
SSA: Sub-Saharan Africa
